# Comparative efficacy and safety of traditional Chinese patent medicine for cognitive dysfunction in diabetic cognitive dysfunction: A protocol for systematic review and Bayesian network meta-analysis

**DOI:** 10.1097/MD.0000000000028946

**Published:** 2022-03-11

**Authors:** Kai Wang, Zhenyuan Jiang, Xiaowen Yu, Yuze Shao, Hailiang Liu, Susu Wu, Linghui Kong, Zhonglin Wang

**Affiliations:** a Shandong Provincial Hospital Affiliated to Shandong First Medical University, China; b Affiliated Hospital of Shandong University of Traditional Chinese Medicine, Jinan, Shandong Province, China; c First College of Clinical Medicine, Shandong University of Traditional Chinese Medicine, China.

**Keywords:** diabetic cognitive dysfunction, network meta-analysis, protocol, traditional Chinese patent medicine

## Abstract

**Background:**

More and more studies have shown that cognitive dysfunction is one of the main complications of diabetes. The disorder of glucose and lipid metabolism seriously damages brain function and accelerates the conversion to dementia. At present, there are no drugs that can directly treat diabetic cognitive dysfunction. All drugs for the treatment of this disease achieve the purpose of treatment through strict control of blood sugar levels. This method has great limitations. Traditional Chinese patent medicines (TCPMs) work through multiple targets and multiple pathways, which can not only effectively correct the state of glucose and lipid metabolism disorders, but also significantly improve cognitive ability, but there is a lack of systematic evaluation of their effectiveness and safety. We use the method of network meta-analysis to systematically and comprehensively compare the effectiveness and safety of different Chinese patent medicines.

**Methods:**

We will comprehensively search the following databases, including Web of Science, PubMed, The Cochrane Library, EMBASE, China National Knowledge Infrastructure, Chinese Scientific Journals Database, Wanfang database and China BioMedical Literature. We will include all randomized controlled trials that meet the inclusion criteria, starting from the establishment of the database until September 2021. Two researchers will independently screen the literature based on inclusion criteria. While extracting data, we also assess the risk of bias in the included studies. All the data and evidence obtained will be evaluated by the method of Bayesian network meta-analysis.

**Results:**

This study will evaluate the effectiveness and safety of various TCPMs for diabetic cognitive dysfunction.

**Conclusion:**

The results of this study will provide valuable references for the clinical application of TCPMs, and assist clinicians in formulating more reasonable diagnosis and treatment strategies.

**Ethics and dissemination:**

This study does not require ethical approval.

**International Platform of Registered Systematic Review and Meta-analysis Protocols registration number:**

INPLASY202190008.

## 1 Introduction

Diabetes mellitus is a metabolic disease characterized by chronic and persistent elevated blood sugar.^[1]^ With the development of economy and society, the change of lifestyle, and the advancement of the aging of the population, the incidence of diabetes mellitus continues to increase. Statistics from the International Diabetes Federation show that, by the end of 2019, the number of diabetic patients in China had reached 116.4 million, and the direct economic loss was as high as US$109 billion.^[2]^ At the same time, patients with type 2 diabetes often have diseases of the heart, brain, and kidney systems.^[3,4]^ Among them, cognitive dysfunction is one of the more common ones, the clinical manifestations are decreased learning and memory abilities, and the impairment of spatial positioning and motor coordination is particularly obvious.^[5]^ At present, the Simple Mental State Examination Scale and the Montreal Cognitive Assessment Scale (MoCA) are often used to assess the cognitive state of patients.^[6,7]^ A survey of 3246 people over 60 years old showed that the prevalence of diabetes with mild cognitive impairment was 21.8%.^[8]^ Hyperglycemia can also promote cognitive impairment.^[9]^ Based on this, some experts believe that, similar to diabetic foot and diabetic retinopathy, cognitive dysfunction is one of the main complications of diabetes.^[10]^

Being in a state of high blood sugar for a long time will have a very obvious impact on the structure and function of the brain. Compared with nondiabetic patients of the same age, the incidence of diabetic patients with reduced cerebral blood flow, brain atrophy, white matter disease, and cerebral microvascular disease has increased significantly.^[11–13]^ Functional MRI diffusion tensor imaging of diabetic patients found: The connecting fibers between the brain regions are demyelinated, and there is obvious white matter damage in diabetic patients.^[14,15]^ A study from the Netherlands used a moving cube algorithm to perform three-dimensional reconstruction to calculate the different morphological characteristics of the white matter of the brain shows that compared with the control group, the brain areas closely related to cognitive functions such as the frontal lobe and hippocampus of the type 2 diabetes group have a higher eccentricity rate.^[16]^

The above studies have shown that the metabolic disorder of diabetic patients will directly damage the brain tissue, leading to cognitive impairment and accelerating the speed of conversion to dementia, but its specific mechanism has not yet been fully elucidated. The blood–brain barrier (BBB) is an important structure to maintain the stability of the brain environment, and it is a functional complex composed of blood vessels and nerves.^[17]^ Some scholars believe that the damage of BBB is one of the causes of cognitive dysfunction in diabetes. Persistent glucose and lipid metabolism disorders in diabetic patients,^[18]^ as well as the long-standing inflammatory state, destroy the microvascular structure, decrease the function of the BBB, impede the transport of nutrients, and reduce the elimination of harmful metabolites in the brain, and the brain tissue is further affected. Violation promotes the generation of cognitive dysfunction. The persistent high glucose state in diabetic patients promotes the production of large amounts of reactive oxygen species and nonenzymatic glycosylation products.^[19,20]^ It causes damage to the temporal lobe, especially the hippocampal neurons, and significantly reduces the patient's memory. In addition, abnormal insulin signal transduction, imbalance of central nervous system Ca^2+^ homeostasis, imbalance of intestinal flora, etc are all important pathological mechanisms of cognitive dysfunction in diabetes.^[21–23]^

Early detection and intervention of diabetic cognitive dysfunction has important clinical significance. Studies have shown that liraglutide can alleviate neuropathological changes by improving insulin resistance, thereby improving the cognitive function of diabetic mice to a certain extent.^[24]^ Studies have also shown that drugs such as glibenclamide and simvastatin can reduce neuroinflammation in hippocampus and inhibit oxidative stress, thereby improving the cognitive function of diabetic rats.^[25]^ Some scholars have conducted a systematic review of intervention drugs and methods for this disease, and found that all studies are based on strict control of blood glucose levels. There is currently no specific treatment that can delay the process of cognitive impairment in diabetic patients.^[26]^ The American Academy of Neurology also recommends cognitive function training as a useful attempt to improve the cognitive function of diabetic patients, but the effectiveness of this method needs to be further confirmed.^[27]^

Traditional Chinese patent medicine is the essence of Chinese medicine and a powerful weapon for Chinese medicine to prevent and treat diseases. After long-term clinical practice, it has been found that Chinese patent medicine has many advantages such as definite curative effect, long-lasting efficacy, few side effects, and high safety. Commonly used Chinese patent medicines for the treatment of diabetes include Xiaoke Pills, Jinqi Jiangtang Tablets, Yuquan Capsules, Qizhi Jiangtang Capsules, etc. Numerous clinical studies and systematic reviews have confirmed that proprietary Chinese medicines can improve the cognitive ability of diabetic patients. Basic research shows that the monomeric active ingredients of many Chinese herbal medicines can effectively improve the cognitive function of diabetic model animals: gastrodin can improve the cognitive function of diabetic rat models by inhibiting endoplasmic reticulum stress and NLRP3 inflammasome activation^[28]^; ginsenosides can effectively block the development of diabetic cognitive dysfunction by acting on the STAT5-PPARγ pathway and the PI3K/Akt pathway.^[29]^ There are many kinds of Chinese patent medicines for the treatment of this disease. The network meta-analysis (NMA) can compare the efficacy and safety of different Chinese patent medicines in the treatment of diabetic cognitive dysfunction, and provide comprehensive and conclusive evidence. Compared with traditional meta-analysis, it has obvious advantages which can provide reliable evidence for clinical decision-making.

## 2 Methods and analysis

We will use Bayesian NMA. Then we compliant PRISMA-P guidelines to conduct this study.

### 2.1 Study registration

This NMA has been registered on the International Platform of Registered Systematic Review and Meta-analysis Protocols and the registration number is: INPLASY202190008 (URL = https://inplasy.com/inplasy-2021-9-0008/).

#### 2.1.1 Inclusion criteria

We will include all randomized controlled trials that use proprietary Chinese medicines to treat diabetic cognitive dysfunction, as well as related clinical trials, for example, I/II early stage, stage III trial, prospective and retrospective observational studies; we will exclude meta-analysis, case reports, and studies with insufficient data. The language is limited to Chinese and English.

#### 2.1.2 Participants

The diagnosis of diabetic cognitive dysfunction will be based on the diagnostic criteria of the “Chinese Type 2 Diabetes Prevention and Control Guidelines (2017 Edition)” and the MoCA score <26 points. Factors such as age, race, and disease course are not restricted.

#### 2.1.3 Interventions

The experimental group was treated with traditional Chinese medicine combined with conventional Western medicine, including Xiaoke Pill, Jinqi Jiangtang Tablet, Yuquan Capsule, Qizhi Jiangtang Capsule, etc; the control group received conventional Western medicine treatment, including oral medication or insulin injection. Randomized controlled trials that use 2 or more proprietary Chinese medicines or combined acupuncture, moxibustion, and other traditional Chinese medicine methods are excluded.

#### 2.1.4 Outcomes

According to the MoCA, a 5-level scoring method of 0 to 4 points is adopted. The main indicators are: total clinical effectiveness, blood glucose stability, and improvement of cognitive function. Secondary indicators include relapse rate, the degree of stability of glycosylated hemoglobin, and the improvement rate of visual space function. The included literature must cover one or more main indicators.

### 2.2 Database and search strategy

We will search PubMed, Cochrane Library, ClinicalTrials, Embase, Chinese national knowledge infrastructure database, Weipu database, Wanfang database, China Biomedical Database. The Chinese search terms are “diabetic cognitive dysfunction”, “diabetic dementia”, “Chinese patent medicine”, and “randomized controlled trial”. The English search terms are “traditional Chinese patent medicine”, “TCPM”, “Diabetic cognitive dysfunction”, “Diabetic cognitive impairment”, “Randomized controlled”. The search time limit is from the establishment of each database to September 2021. (The retrieval scheme of the PubMed database is listed in Table 1).

**Table 1:** Detailed search strategy for PubMed.

No.	Search item
#1	“Diabetes Mellitus”[Mesh]
#2	(((Diabetes Mellitus, Type 2[MeSH Terms]) OR (Diabetes Mellitus, Type 1[MeSH Terms])) OR (Diet, Diabetic[Title/Abstract])) OR (Glucose Intolerance[Title/Abstract])
#3	#1 OR #2
#4	Complementary medicine [Title/Abstract] OR medicine, alternative [Title/Abstract] OR Traditional Chinese patent medicine [Title/ Abstract] OR Chinese proprietary medicine [Title/Abstract]
#5	Xiaoke pill [Title/Abstract] OR Yuquan capsule [Title/Abstract] OR Qizhi Jiangtang Capsules [Title/Abstract] OR Jinqi Jiangtang Tablets [Title/Abstract] OR Liuwei Dihuang capsule [Title/Abstract] OR Shenqi jiangtang capsule [Title/Abstract] OR Jinlida Particles [Title/Abstract] OR Yangyin jiangtang capsule [Title/Abstract]
#6	#4 OR #5
#7	(Randomized controlled trial) [Publication Type] OR (Controlled clinical trial [Publication Type])
#8	(Randomized [Title/Abstract]) OR (random allocation [Title/Abstract])
#9	#7 OR #8
#10	#3 AND #6 AND #9

### 2.3 Study selection and data extraction

Two investigators independently screened the articles according to the inclusion and exclusion criteria, and cross-checked them. If there is a disagreement, they will discuss and negotiate with the third investigator to make a ruling. The main data extracted include: the basic information of the included study (first author, research title, sample size, year, age, course of disease, treatment course); key elements of bias risk evaluation; baseline characteristics and intervention measures of the research object; and outcome indicators.

### 2.4 Risk of bias assessment

Two researchers will independently assess the quality of each trial based on the Cochrane Risk of Bias Risk Assessment Tool recommended by Cochrane Handbook version 5.1.0. Use the decision words “high risk”, “low risk”, and “unclear risk” to evaluate the quality of the input article in 7 aspects. Bias risk: whether the random sequence is sufficient; whether there is hidden allocation; whether blind method is used; whether the result data is complete; whether there is selective reporting; whether there is publication bias; others.

### 2.5 Statistical analysis

We will use Stata 14.0 software and Markov chain–Monte Carlo method to conduct Bayesian meta-analysis. Three Markov chains will be used for simulation, and the number of iterations will be set at 50,000 (the first 20,000 are used for annealing to eliminate the effect of the initial value, and the last 30,000 are used for sampling).

The reticular diagram will be drawn by Stata 15.0 software to show the direct and indirect comparison between different interventions. The relative odds ratio (OR) and its 95% confidence interval (CI) are calculated to evaluate the consistency of each closed loop. The lower limit of 95% CI is equal to 1, indicating good consistency. If relative OR is close to 1, direct evidence and indirect evidence are consistent, and the fixed effect model is adopted for analysis. Otherwise, the closed-loop is considered to have obvious inconsistencies, and the random effect model is used for analysis. Dichotomous data will be represented by OR and 95% CI, and *P* < .05 was considered statistically significant. WinBUGS 1.4.3 will be used to rank the efficacy of different interventions and the area under the curve will be recorded (the area under the curve will be expressed as a percentage, the larger the value, the better the effect).

### 2.6 Assessment of heterogeneity

If (*P* > .10 and I^2^ < 50%), we will use the fixed-effect model. Otherwise, we will further explore the source of heterogeneity, and if the source cannot be found, the random-effects model will be used for analysis.

### 2.7 Subgroup analysis and sensitivity analysis

If the information is sufficient, subgroup analysis will be considered. Carry out a sensitivity analysis based on the symptom improvement rate and evaluate the clinical similarity and methodology of the included studies to determine the reliability of the results of this study.

### 2.8 Evaluation of publication bias

The total clinical effective rate, the degree of stability of blood glucose, the degree of improvement of cognitive function, the degree of stability of glycosylated hemoglobin, and the improvement rate of visual space function are indicators. The effect size of each study is used as the abscissa, and the standard error of the effect size is taken as the ordinate draws an inverted funnel chart. If the inverted funnel chart is basically symmetrical, it indicates that the study has a small sample effect or publication bias is less likely.

### 2.9 Grading the quality of evidence

We will use GRADE^[30]^ to evaluate the quality of evidence from the following 5 aspects: risk of bias, indirectness, inconsistency, imprecision, and publication bias.

## 3 Discussion

With the development of economy and society, the incidence of metabolic diseases such as diabetes remains high, and the metabolic disorders of sugar and lipids seriously damage brain function. If effective treatment is not available, the cognitive ability of the patient will decrease, and the transformation process of elderly patients with cognitive dysfunction to dementia will be significantly accelerated, which will bring a heavy economic burden to the whole society. There is currently no drug specifically for the treatment of diabetic cognitive dysfunction. Chinese patent medicine are the essence of Chinese medicine. Under the strict guidance of the national drug regulatory authority, a rigorous formulation, definite curative effect, and long-term clinical use of prescriptions with no adverse reactions are selected, and they are made into various dosage forms that are easy to take through scientific preparation processes. It has good compliance and treatment effect. This study uses the method of network mate analysis to evaluate the advantages and disadvantages of various Chinese patent medicines for the treatment of cognitive dysfunction in diabetes, and to provide reasonable evidence support for clinicians’ decision-making. The quality of the analysis may depend on the equality of insufficient data, such as possible publication bias in qualified literature. Therefore, in future studies, we will include more high-quality, multi-center clinical studies and evidence to evaluate the effectiveness and safety of Chinese patent medicines in the treatment of diabetic cognitive dysfunction.

## Author contributions

**Conceptualization:** Kai Wang, Zhenyuan Jiang.

**Data curation:** Kai Wang, Xiaowen Yu.

**Formal analysis:** Kai Wang, Zhenyuan Jiang, Xiaowen YU, Yuze Shao, Linghui Kong, Zhonglin Wang.

**Funding acquisition:** Kai Wang, Hailiang Liu.

**Investigation:** Zhenyuan Jiang, Yuze Shao, Hailiang Liu.

**Methodology:** Kai Wang, Zhenyuan Jiang, Yuze Shao.

**Project administration:** Kai Wang, Zhonglin Wang.

**Resources:** Susu Wu.

**Software:** Kai Wang, Zhenyuan Jiang, Linghui Kong.

**Supervision:** Kai Wang, Susu Wu, Zhonglin Wang.

**Validation:** Kai Wang, Yuze Shao, Linghui Kong.

**Visualization:** Xiaowen YU, Susu Wu, Linghui Kong.

**Writing – original draft:** Kai Wang, Zhenyuan Jiang.

**Writing – review & editing:** Zhonglin Wang.
